# Mescaline Alters Cerebellar Function, Global Connectivity, and Frequency-Selective Acoustic Gating: A BOLD fMRI Study in Awake Rats

**DOI:** 10.1007/s12264-026-01632-3

**Published:** 2026-05-21

**Authors:** Noah Cavallaro, Priya Rai, David Akins, Sima Soltanpour, Md Taufiq Nasseef, Richard J. Ortiz, Rachel Utama, Caitlyn R. Cody, Anoushka Mistry, Heather C. Brenhouse, Praveen P. Kulkarni, Craig F. Ferris

**Affiliations:** 1 Center for Translational Neuroimaging, Northeastern University, Boston, MA 02115 USA; 2 School of Information Technology, Carleton University, Ottawa, ON K1S 5B6 Canada; 3 Department of Mathematics, College of Science and Humanity Studies, Prince Sattam Bin Abdulaziz University, 16273 Riyadh, Saudi Arabia; 4 Department of Psychology, Northern Illinois University, DeKalb, IL 60115 USA; 5 Psychology Department, Northeastern University, Boston, MA 02115 USA; 6 Departments of Psychology & Pharmaceutical Sciences, Northeastern University, Boston, MA 02115 USA

**Keywords:** Hippocampus, Cerebellum, BOLD resting state functional connectivity, Pharmacological MRI, Reward, Fear

## Abstract

Mescaline, a 5-HT2A agonist psychedelic used ceremonially for millennia, lacks neuroimaging characterization due to its Schedule 1 status. Using pharmacological and resting-state fMRI in awake rats, we report mescaline's first comprehensive neurobiological profile. Acutely, mescaline produced cerebellar-selective BOLD suppression, suggesting functional disconnection from forebrain structures. Paradoxically, resting-state analysis revealed global hyperconnectivity, with the cerebellum forming enhanced connections to the hippocampus, thalamus, somatosensory cortex, and midbrain. Mescaline abolished normal BOLD responses to rewarding olfactory stimuli, indicating disrupted sensory processing. Pre-pulse inhibition showed frequency-dependent acoustic gating effects: enhancement at 4 kHz (+ 27.6%) and 20 kHz (+ 27.3%), but impairment at 12 kHz (− 16.4%). These findings distinguish mescaline from LSD and psilocybin, implicating the cerebellum as a dysregulated sensory filter that floods forebrain circuits with unprocessed sensorimotor information—a potential mechanism underlying psychedelic-induced perceptual alterations.

**Supplementary Information:**

The online version contains supplementary material available at https://doi.org/10.1007/s12264-026-01632-3.

## Introduction

Mescaline (3,4,5-trimethoxyphenethylamine), a naturally occurring psychedelic compound derived from cacti such as peyote (*Lophophora williamsii*) and San Pedro (*Echinopsis pachanoi*), represents one of humanity's oldest documented psychoactive substances [1]. Archaeological evidence and carbon dating reveal its ceremonial use across the Americas spanning over thirty-five hundred years [2], making it among the longest-studied psychedelics in human history. Despite this extensive cultural legacy, scientific understanding of mescaline's neurobiological mechanisms remains remarkably limited due to its classification as a Schedule 1 controlled substance in the early 1970s [3].

Contemporary indigenous use of mescaline-containing cacti in Native American religious ceremonies provides compelling evidence for its safety profile and therapeutic potential. Comprehensive neuropsychological assessments of Native American Church members reveal no adverse cognitive outcomes associated with lifetime peyote use, while anonymous online surveys document self-reported psychiatric improvements, including mystical experiences and ego dissolution [4, 5]. This real-world evidence of safety and efficacy has promoted renewed scientific interest in mescaline's therapeutic applications.

The recent landmark study by Klaiber and colleagues represents a critical milestone in mescaline research, providing the first double-blind, placebo-controlled investigation in healthy volunteers [6]. Their dose-dependent findings demonstrated manageable autonomic effects (increased blood pressure, heart rate, and body temperature) alongside the characteristic psychedelic phenomena of ego dissolution and altered visual perception. Importantly, mescaline exhibited a comparable safety profile to established psychedelics like LSD and psilocybin [7], reinforcing its potential as a therapeutic agent for psychiatric disorders [8]. However, this foundational work highlighted a crucial knowledge gap: the complete absence of neuroimaging data characterizing mescaline's direct effects on brain activity and functional connectivity.

Mescaline belongs to the serotonergic psychedelic class and shares the primary mechanism of 5-HT2a receptor activation with LSD and psilocybin [9]. This pharmacological similarity has led researchers to extrapolate findings from LSD and psilocybin studies to predict mescaline's neural effects [10–12]. However, such extrapolations may be misleading, as accumulating evidence suggests each psychedelic exhibits distinct neurobiological signatures despite shared receptor targets. Neuroimaging studies of psilocybin have revealed a characteristic pattern of decreased default mode network connectivity coupled with increased global brain integration, resulting in a more flexible and interconnected neural state [13]. This "neural desynchronization" manifests as reduced functional connectivity between visual and sensorimotor networks, potentially explaining the visual distortions characteristic of psychedelic experiences [14]. Importantly, psilocybin also triggers rapid neuroplasticity changes, enhancing synaptogenesis and dendritic growth within 24 hours of exposure [15], complicating interpretation of acute versus sustained effects in human studies.

Recent advances in awake animal neuroimaging have begun to overcome the limitations inherent in human psychedelic research, including prior drug exposure, unblinded conditions, and confounding neuroplasticity effects. Ghaw and colleagues demonstrated that LSD produces dose-dependent effects on brain functional activity in awake rats, with lower doses primarily affecting cortical regions and higher doses engaging thalamic and cerebellar circuits [16]. Their finding of dose-dependent increases in negative BOLD signal contrasts sharply with our recent psilocybin study, which revealed global increases in positive BOLD signal and pronounced cerebellar hyperconnectivity [17]. These divergent patterns underscore the importance of characterizing each psychedelic individually rather than assuming mechanistic equivalence. Based on our previous findings with LSD and psilocybin in awake rats, we hypothesized that mescaline would produce region-specific alterations in BOLD signal, accompanied by enhanced global functional connectivity. However, we anticipated that the specific pattern of activation would distinguish mescaline from other psychedelics, potentially revealing novel mechanisms of psychedelic action.

## Materials and Methods

### Animals

Adult male (*n =* 12) and female (*n =* 12) Sprague-Dawley rats, approximately 100 days of age, were purchased from Charles River Laboratories (Wilmington, MA, USA). The sample size was determined based on previous awake rat fMRI studies from our laboratory [16–19]. Animals were housed by sex in Plexiglas cages (two per cage) and maintained in ambient temperature (22–24°C). Animals were maintained on a reverse L-D cycle with lights off at 09:00 AM and studied during the dark phase when they are normally active. All experiments were conducted between 10:00 AM and 6:00 PM to avoid the transitions between the L-D dark cycles. Food and water were provided *ad libitum*. All animals were acquired and cared for in accordance with the guidelines published in the NIH Guide for the Care and Use of Laboratory Animals. All methods and procedures described below were pre-approved by the Northeastern University Institutional Animal Care and Use Committee under protocol numbers 23-0407R. Northeastern University’s animal care and use program and housing facilities are fully accredited by AAALAC, International. The protocols used in this study followed the ARRIVE guidelines for reporting *in vivo* experiments in animal research [20]. Animals were monitored daily over the duration of the study for general health, food, and water consumption. A 15% loss in body weight was set as a humane endpoint.

### Drugs

**Fig. 1:**
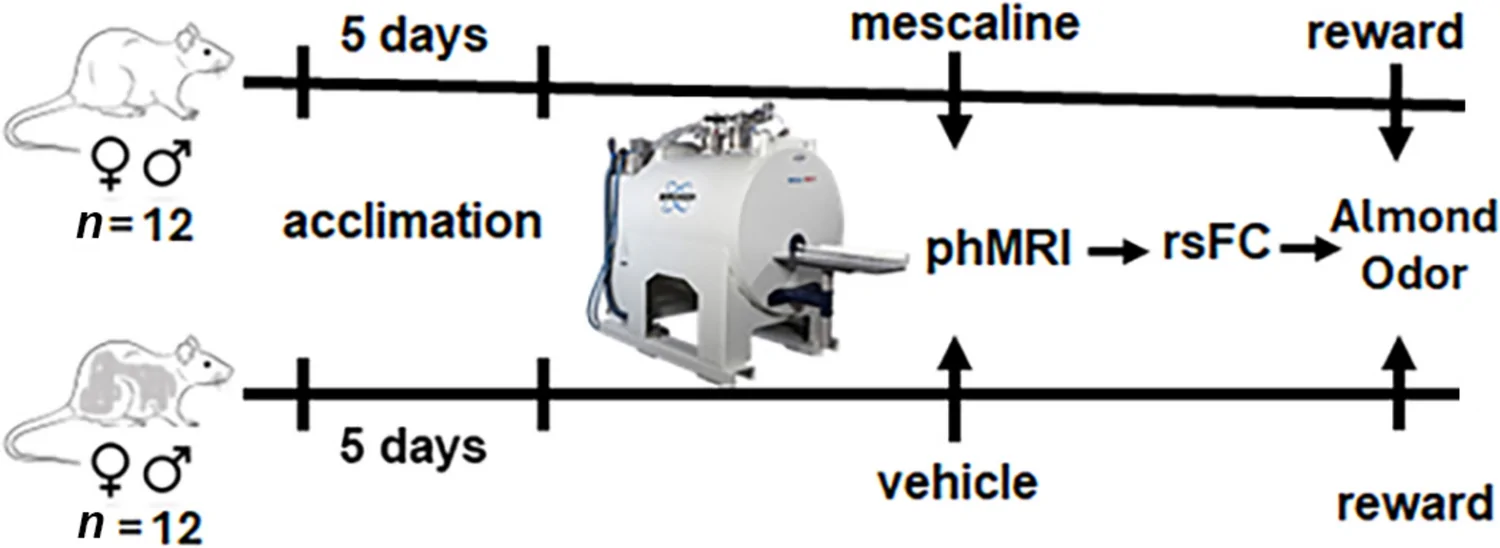
Experimental protocols. Shown is a diagram of the timeline of the sequence of experimental protocols.

Mescaline was acquired through the National Institute on Drug Abuse (NIDA) and distributed by the Research Triangle Institute. Mescaline was prepared in sterile saline (0.9% NaCl) for a dose of 50 mg/kg. Rats were divided into two experimental groups of 12 each, with equal numbers of males and females. The schedule of experimental procedures is shown in Fig. 1. After acclimation (see below), rats were given mescaline or a vehicle while fully awake during the scanning session. The dose of 50 mg/kg for rats was taken from the literature [21, 22].

### Acclimation for Awake Imaging and Image Acquisition

To mitigate the stress associated with head restraint, rats were acclimated to the restraining and imaging protocol as previously described [23, 24]. These acclimation sessions were conducted daily for five consecutive days. This acclimation protocol has been previously validated to significantly reduce autonomic and somatic signs of stress, including respiration rate, heart rate, motor activity, and plasma corticosterone levels, leading to improved fMRI signal stability [25].

The animal setup and imaging protocol have been described in detail in previous publications [16, 18, 19, 26]. Supplementary Table S1 lists all of the imaging parameters for each of the imaging protocols used in the study and described below. In brief, five to six rats were imaged in a day. Each day had a mixture of the different experimental groups known to all the investigators. Imaging sessions were conducted under dim red illumination. Radio frequency signals were sent and received with a quadrature volume coil built into the animal restrainer (Ekam Imaging, Boston, MA, USA) [27]. Imaging sessions were conducted using a Bruker Biospec 7.0 T/20-cm USR horizontal magnet (Bruker, Billerica, MA, USA) and a 2 T/m magnetic field gradient insert (ID = 12 cm) capable of a 120-μs rise time. At the beginning of each imaging session, a high-resolution anatomical data set was collected using a rapid acquisition, relaxation enhancement pulse sequence (RARE factor 8); (25 slices; 1 mm; field of view (FOV) 3.0 cm^2^; data matrix 256 × 256; repetition time (TR) 3 s; echo time (TE) 12 ms; Effective TE 48 ms; number of excitations (NEX) 3; 4.48 min acquisition time; in-plane resolution 117.2 μm^2^). The sequence of functional imaging protocols is shown in Fig. 1. Pharmacological MRI (phMRI) involved a five min baseline of 10 image acquisition/min, then mescaline treatment followed by 20 min of 200 image acquisition for a total of 25 min of scan time. Resting state functional MRI (rsFC) was performed approximately 10 min after the phMRI protocol. This scanning protocol lasted 15 min. Odor presentation of almond occurred approximately 5 min after rsFC and consisted of a five min baseline (50 image acquisitions) followed by odor presentation for five min and another 5 min after cessation of odor for a total scanning time of 15 min or 150 image acquisitions.

The phMRI images were captured using a multi-slice Half Fourier Acquisition Single Shot Turbo Spin Echo (HASTE) pulse sequence. This involved collecting 22 slices, each 1.1 mm thick, with the same FOV of 3.0 cm^2^. The data matrix was 96 × 96, with a TR of 6 seconds, TE of 3.75 milliseconds, and an effective TE of 22.5 milliseconds. The acquisition took approximately 25 minutes, with an in-plane resolution of 312.5 μm^2^. The odor-provocation images were collected using the same pulse sequence described for phMRI but with a different time course. It should also be emphasized that high neuroanatomical fidelity and spatial resolution are critical in identifying distributed neural circuits in any animal imaging study. Many brain areas in a segmented rat atlas have in-plane boundaries of less than 400 μm^2^ and may extend for over 1000 μm in the rostral/caudal plane. With the development of a segmented, annotated 3D MRI atlas for rats (Ekam Solutions, Boston, MA, USA), it is now possible to localize functional imaging data to precise 3D “volumes of interest” in clearly delineated brain areas. This spatial resolution was sufficient to identify the bilateral habenula, with approximately 4–5 voxels on each side, but not to differentiate between the lateral and medial habenula.

The rsFC images were collected using a spin-echo triple-shot EPI sequence (imaging parameters: matrix size = 96 × 96 × 20 (height × width × depth), TR/TE=1000/15 ms, voxel size = 0.312 mm × 0.312 mm × 1.2 mm, slice thickness = 1.2 mm, with 200 repetitions, time of acquisition 15 min.

### Preprocessing Pipeline for Resting-State fMRI Data

Resting-state fMRI data underwent our established standardized preprocessing pipeline following our previous studies [19, 26]. This included skull-stripping, motion correction and censoring, spatial normalization, smoothing, temporal filtering, and nuisance regression to yield cleaned BOLD time series for connectivity analysis.

The preprocessing of rat fMRI data was conducted using integrated tools from Analysis of Functional NeuroImages (AFNI_17.1.12), the FMRIB Software Library (FSL, v5.0.9), Deformable Registration *via* Attribute Matching and Mutual-Saliency Weighting (DRAMMS 1.4.1), Statistical Parametric Mapping (SPM12), and custom MATLAB (R2023a) scripts. The complete workflow, executed below, is designed to mitigate noise from physiological sources and motion artifacts, which are critical in awake animal imaging.

For initial Quality Control and Skull-Stripping, each subject's brain tissue masks were manually delineated on the mean functional image using 3DSlicer (version 5.2.2) and applied to remove non-brain tissue. For motion outlier detection and scrubbing, rigorous motion censoring was performed. Time points corrupted by substantial motion were identified as outliers using AFNI’s @1dDiffMag and 3dToutcount functions. The threshold for defining a "bad" time point was set at a frame-wise displacement (FD) > 0.3 mm, calculated from the six motion parameters. Any time point exceeding this threshold, as well as the one preceding and two following it, was flagged for subsequent regression. The mean FD did not differ significantly between vehicle (0.12 ± 0.02 mm) and mescaline (0.14 ± 0.03 mm) groups (*P =* 0.47, two-sample *t*-test), and no entire subject dataset was excluded based on these criteria. For slice Timing Correction, temporal correction of interleaved slice acquisition was applied using SPM12 to adjust for differences in slice acquisition times. For head Motion Correction, rigid-body realignment for head motion correction was performed using FSL’s MCFLIRT, with the first volume serving as the reference image. The six estimated motion parameters (three rotations, three translations) were retained for later nuisance regression. For spatial Normalization, all images were aligned and registered to the commercially available, segmented 3D MRI Rat Brain Atlas (© Ekam Solutions, Boston, MA, USA; https://ekamsolutions.com/mri-atlases/rat-brain-atlases/), which parcellates the brain into 169 discrete, annotated regions of interest (ROIs) using affine registration *via* DRAMMS to ensure anatomical consistency across subjects. For spatial smoothing, normalized images were spatially smoothed using a Gaussian kernel with a full width at half maximum (FWHM) of 0.8 mm (approximately 2.5 times the voxel dimension) to improve the signal-to-noise ratio while preserving anatomical specificity. For temporal filtering, a band-pass filter (0.01 Hz to 0.1 Hz) was applied to the time series of each voxel using AFNI’s 3dBandpass to restrict analysis to frequencies relevant for slow, spontaneous BOLD fluctuations while reducing high-frequency physiological noise and low-frequency drift. Finally, a comprehensive nuisance regression step was implemented within the general linear model (GLM) framework in SPM12. The following regressors were included to remove spurious variance: (1) The six motion parameters and their first temporal derivatives (12 regressors total). The flagged motion outlier time points (scrubbing regressors). (2) The mean time series from white matter and cerebrospinal fluid (CSF) masks, derived from the atlas segmentation 36, to account for physiological noise from non-neural sources. The residual time series from this nuisance regression model, representing the BOLD signal with the effects of the above confounds removed, were used for all subsequent functional connectivity analyses.

Note that the global mean signal was not regressed out, as our primary analysis focuses on absolute correlation values for degree centrality. The potential confounding effects of global signal were assessed in supplementary analyses using global signal regression (GSR), which did not alter the pattern of significant group differences in hyperconnectivity.

The region-to-region functional connectivity analysis was conducted to measure the correlations in spontaneous BOLD fluctuations. In this analysis, a network consists of nodes (brain regions of interest or ROIs) and edges (connections between regions). We averaged the voxel time series data within each node based on the residual images obtained through the nuisance regression procedure. Pearson’s correlation coefficients were computed across all pairs of nodes (14, 196 pairs) for each subject within all three groups to assess interregional temporal correlations. The resulting *r*-values, ranging from −1 to 1, were Z-transformed using Fisher's Z transform to improve their normality. We constructed 169 × 169 symmetric connectivity matrices, with each entry representing the strength of an edge. Group-level analysis was then conducted to examine functional connectivity in the experimental groups. The Z-score matrices obtained from one-group *t*-tests were clustered using the K-nearest neighbors clustering method to identify how nodes cluster together and form resting-state networks. A Z-score threshold of |Z| = 2.3 was applied to eliminate spurious or weak node connections for visualization purposes.

### Data Analysis

Prior to phMRI analysis, rats were screened for motion artifact. Any subject with motion exceeding the size of a functional voxel (~300 µm) in any orthogonal direction was eliminated from the study. We maintain motion to under half a voxel size (~150 µm) for inclusion. Both vehicle and mescaline groups met this criterion, and no subjects were excluded. This was particularly important given the head twitch effect caused by 5HT2a agonists. The results are presented in Supplementary Fig. S1. The time course plots account for head motion in three orthogonal directions for both vehicle and mescaline and show the mean motion in microns ± SE. The size of a functional voxel is ~300 µm. Any subject with motion exceeding this would be eliminated from the study [28, 29]. No subjects were eliminated from the study. It is our practice to try to hold our motion to under one half of a voxel size or approximately 150 µm [26, 30].

A detailed description of the data analysis for functional changes in the BOLD signal following drug challenge in rats is published [16, 26, 31, 32]. In brief, the fMRI data analysis consisted of three main steps: pre-processing, processing, and post-processing. All these steps were executed using SPM-12 (available at https://www.fil.ion.ucl.ac.uk/spm/) and in-house Matlab software. The processing step involved aligning the data to a rat atlas, followed by segmentation and statistical analysis. To achieve registration and segmentation, all functional images were aligned and registered to the 3D MRI Rat Brain Atlas© using the vendor-provided EVA (Ekam Visualization and Analysis; https://ekamsolutions.com/softwares/ ) software (Ekam Solutions, Boston, MA). This atlas provides a pre-segmented template with 169 anatomical regions, which was used to automatically extract time series data from each corresponding region for each subject.

Statistical *t*-tests were performed on each voxel (~ 36,000 voxels in the whole brain) of each subject within their original coordinate system. Before statistical testing, a baseline signal change threshold of 1% was applied to identify voxels with potentially meaningful signal changes. This excluded voxels that never exceeded ±1% BOLD signal change from baseline during the post-injection period. This 1% cutoff was chosen as a conservative floor to eliminate very small fluctuations that likely reflect noise or minor physiological drift, ensuring that only voxels with at least a minimal response were considered for significance testing. Importantly, this thresholding did not automatically deem 1% as significant; it was simply a preprocessing step. All voxels meeting the threshold were then subjected to statistical analysis using a one-sample *t*-test against zero change. The reference for these *t*-tests was a null hypothesis of zero change from baseline. The *t*-test statistics used a 95% confidence level (*P <* 0.05), two-tailed distributions, and heteroscedastic variance assumptions. As a result of the multiple *t*-test analyses performed, a false-positive detection controlling mechanism was introduced. We set the acceptable false discovery rate at Q=0.05 (5%). After performing voxel-wise tests, a False Discovery Rate (FDR) correction (using the Benjamini–Hochberg procedure) across all voxels in the brain was applied to control for multiple comparisons. This subsequent filter guaranteed that the false-positive detection rate was below our cutoff of 0.05. The formulation of the filter satisfied the following expression:

$${P}_{i}\le \frac{i}{V}\frac{q}{c\left(V\right)} ,$$

In the equation, Pi represents the *P*-value derived from the *t*-test conducted at the i-th pixel within the region of interest (ROI), comprising V pixels, with each pixel ranked according to its probability value. For our analysis, we set the false-positive filter value q at 0.2, and we fixed the predetermined constant c(V) at unity, following a conservative approach for assessing significance [33]. Pixels that achieved statistical significance retained their relative percentage change values, while all other pixel values were set to zero. Our analysis employed a 95% confidence level, two-tailed distributions, and assumed heteroscedastic variance for the *t*-tests. In the post-processing phase, we compared the number of activated voxels in each of the 169 brain areas between control and mescaline using a matched Wilcoxon signed-rank test statistic. The data were ranked in order of significance, as detailed in Supplementary Table S1 and  S2.

### Functional Connectivity Analysis.

Functional connectivity matrices were constructed using the preprocessed, nuisance-regressed BOLD time series as described above. For our primary analysis, we used the absolute values of Pearson correlation coefficients to focus on the magnitude of functional coupling between regions, irrespective of correlation sign (i.e., both strongly correlated and strongly anti-correlated activity were considered evidence of a functional connection). This approach is commonly used to assess a region's overall level of integration within the whole-brain network. To ensure the robustness of findings derived from absolute correlations, we conducted supplementary analyses using signed correlation values and a range of proportional sparsity thresholds; these confirmed the same pattern of group differences (Supplementary Fig. S2 A and B).

### Degree Centrality

We conducted all network analysis using Gephi, which is an open-source software for network analysis and visualization [34]. We imported the absolute values of the symmetric connectivity matrices for both psilocybin and vehicle data, treating the edges as undirected networks. Degree centrality analysis measures the number of connections that a particular node has within the entire network. Degree centrality is defined as:

$${\mathbf{C}}_{\mathbf{D}}(\mathbf{j})=\sum_{\mathbf{j}=1}^{\mathbf{n}}{\mathbf{A}}_{\mathbf{i}\mathbf{j}}$$

Here, "n" represents the total number of rows in the adjacency matrix denoted as "A," and the individual elements of the matrix are indicated as "Aij," which signifies the count of edges connecting nodes "*i*" and "*j*".

### Statistics

We conducted all statistical analyses for the graph theory assessment using GraphPad Prism. To decide whether parametric or non-parametric assumptions were appropriate for different group subregions, we performed normality tests. We used Shapiro-Wilk's tests to assess the normality assumption. Subregion degree centrality *P*-values exceeding 0.05 were considered to exhibit a normal distribution. Once the normality assumptions were confirmed, we employed one-way ANOVA to compare the degree of centrality between the experimental groups in various subregions.

### Effect Size

The magnitude of treatment effects was quantified using Omega Squared (ω^2^), an unbiased estimator of the proportion of total variance in the dependent variable accounted for by the independent variable. Omega Squared was computed as:

$${\omega }^{2}=\frac{S{S}_{between}-(k-1)\times M{S}_{within}}{S{S}_{total}+M{S}_{within}}$$

where $S{S}_{between}$ and $S{S}_{total}$ are the between-groups and total sums of squares, $M{S}_{within}$ is the mean square error, and k is the number of groups. This metric is reported alongside *P*-values to provide a measure of practical significance, with values <0.01, <0.06, and <0.14 typically interpreted as small, medium, and large effects, respectively. The use of ω^2^ follows our group’s previous neuroimaging pharmacology studies, e.g. [19, 26].

### Prepulse Inhibition

In a separate study, male (*n =*8) and female (*n =* 8) Sprague-Dawley rats were obtained from Charles River Laboratories (Wilmington, MA) on post-natal day (PND) 70. Pre-pulse inhibition (PPI) experiments were run using the startle reflex hardware package (MED-ASR-PRO1, Med Associates Inc, St. Albans, VT). Rats were placed in foam-lined sound-attenuated startle chambers, which contained an internal animal holder with a grid rod floor on a startle platform containing the load cell, which, along with the load cell amplifier, converts force on the platform to a voltage representing the startle response. Speakers for delivering white noise bursts and pre-pulse tones were positioned 1 inch behind the animal holder. Four different chambers were used and were calibrated to the animal’s weight prior to the experiment. Data was collected *via* the PPI program within the Startle Reflex Package (SOF-826, Med Associates Inc, St. Albans, VT).

### Experimental Procedure

Twenty-four hours prior to testing, animals were weighed, acclimated to the testing room for 20 minutes, and habituated to the startle chamber for 7 minutes with the apparatus turned on. On the day of testing, animals were again allowed to acclimate to the testing room for 20 minutes prior to mescaline or vehicle injections. Thirty minutes following injections, animals were placed into the unlit startle chamber for testing. Testing consisted of three blocks: acclimation, pre-pulse and noise bursts, and noise bursts only. The acclimation period lasted one minute and was immediately followed by the pre-pulse and noise burst block. This block contained 40 trials in total: 24 pre-pulse trials, which consisted of a 70 dB tone presented in one of three possible frequencies (4, 12, or 20 kHz) played 100 ms prior to a 100 dB white noise burst, and 16 white noise burst only trials. The last block was a noise burst only block and consisted of 15 white noise bursts of varying intensities (70, 80, or 90 dB) separated by a 3–8-s intertrial interval (ITI). Animal holders were cleaned with 50% EtOH between each animal. Testing occurred in the dark phase under red light conditions. The data were analyzed with a repeated measures two-way ANOVA.

## Results

### Pharmacological BOLD MRI

After screening for acceptable motion artifact (Supplementary Fig. S1), rats were analyzed for mescaline-induced changes in the volume of activation (VoA), defined as the number of voxels showing a statistically significant BOLD signal change within each of the 169 atlas-defined brain areas in the 3D MRI brain atlas. Table 1 is a truncated list of brain areas (mean ± SE) that show a significant change in positive VoA, i.e., number of positive BOLD voxels, in response to mescaline. Significant positive activation was observed in 26/169 brain areas. The brain areas are ranked in order of their significance using a critical value of *P <* 0.05. Shown are the *P*-values and effect size given as omega square (ω Sq). The false discovery rate (FDR) was *P =* 0.031. Note that in all cases, there was a significant decrease in positive BOLD VoA with mescaline as compared to vehicle. The measure of effect size is highly significant (<0.20) for a majority of brain areas. In Table 2, 33/169 areas displayed a significant change in negative VoA, with an FDR *P =* 0.039. In all cases, mescaline causes a significant increase in negative BOLD VoA as compared to vehicle. Again, a majority of brain areas had highly significant effect sizes. This effect of decreasing positive BOLD and increasing negative BOLD VoA suggests mescaline reduces brain activity. Tables reporting the positive and negative VoA for all 169 brain areas for mescaline are provided in Supplementary Tables S1 and S2.

**Table 1:** Positive BOLD volume of activity (voxel number).

Brain area	Vehicle			Mescaline		*P* Val	ω Sq
	Ave	SE		Ave	SE		
3rd cerebellar lobule	19	7.53	>	2	1.06	0.001	0.624
Simple lobule cerebellum	33	7.26	>	5	2.89	0.001	0.523
9th cerebellar lobule	3	0.92	>	0	0.00	0.003	0.447
10th cerebellar lobule	6	1.74	>	0	0.00	0.003	0.446
Copula of the pyramis	16	5.55	>	1	0.42	0.003	0.437
5th cerebellar lobule	41	11.18	>	5	2.16	0.003	0.434
Crus 1 of ansiform lobule	46	8.94	>	10	4.77	0.003	0.431
Inferior colliculus	55	8.36	>	17	7.34	0.005	0.400
2nd cerebellar lobule	22	7.02	>	5	2.17	0.005	0.390
Lateral geniculate	5	1.77	>	1	0.84	0.006	0.366
Fastigial n. cerebellum	3	1.12	>	0	0.00	0.008	0.337
Paramedian lobule	10	2.25	>	3	1.59	0.009	0.332
Medial geniculate	11	2.29	>	3	1.61	0.010	0.324
1st cerebellar lobule	5	1.63	>	1	0.53	0.011	0.312
Substantia nigra reticularis	19	4.00	>	7	3.41	0.018	0.262
Flocculus cerebellum	3	0.90	>	2	1.47	0.020	0.252
6th cerebellar lobule	32	8.72	>	4	1.78	0.020	0.248
Superior colliculus	38	7.48	>	15	6.25	0.022	0.240
Interposed n. cerebellum	2	1.16	>	0	0.00	0.022	0.240
Crus 2 of ansiform lobule	10	3.53	>	2	2.21	0.027	0.221
Vestibular n.	13	5.20	>	2	0.92	0.032	0.203
Pineal gland	1	0.44	>	0	0.21	0.032	0.202
4th cerebellar lobule	11	5.49	>	2	1.25	0.033	0.199
Habenula n.	8	2.17	>	2	1.27	0.034	0.198
Dentate gyrus dorsal	26	6.85	>	11	6.44	0.036	0.192
Cochlear n.	6	1.88	>	2	0.93	0.046	0.168

Ave, Average; SE, Standard error; *P* val, Probability value; ω Sq, Omega square; n., nucleus.

**Table 2:** Negative BOLD volume of activity (voxel number).

Brain area	Vehicle			Mescaline		*P* val	ω Sq
	Ave	SE		Ave	SE		
10th cerebellar lobule	17	4.10	<	39	1.64	0.000	0.652
Crus 1 of ansiform lobule	151	11.30	<	243	16.46	0.001	0.575
6th cerebellar lobule	75	8.56	<	128	7.97	0.001	0.547
Paramedian lobule	72	6.24	<	109	5.83	0.001	0.526
Copula of the pyramis	30	5.09	<	62	3.49	0.001	0.522
Vestibular n.	65	5.33	<	94	3.95	0.002	0.489
4th cerebellar lobule	66	8.06	<	92	4.23	0.006	0.362
Simple lobule cerebellum	84	13.18	<	138	10.30	0.007	0.370
7th cerebellar lobule	17	3.20	<	27	1.77	0.008	0.381
Medial pretectal n.	1	0.24	<	0	0.00	0.008	0.358
Pineal gland	1	0.37	<	2	0.41	0.009	0.335
Central gray	17	2.27	<	27	2.80	0.011	0.302
5th cerebellar lobule	117	16.63	<	179	9.53	0.011	0.318
3rd cerebellar lobule	66	7.95	<	97	6.95	0.011	0.332
Superior colliculus	93	12.31	<	150	14.93	0.014	0.280
Medial septum	1	0.41	<	5	1.12	0.016	0.264
Lemniscal n.	15	2.84	<	26	3.46	0.018	0.250
Crus 2 of ansiform lobule	79	10.58	<	110	5.74	0.018	0.272
Lateral septal n.	68	12.49	<	121	18.37	0.018	0.250
Raphe magnus	13	0.68	<	9	1.57	0.020	0.292
8th cerebellar lobule	51	6.83	<	68	2.50	0.025	0.270
Cochlear n.	20	2.26	<	28	2.46	0.027	0.219
Substantia nigra reticularis	16	3.38	<	33	6.00	0.027	0.221
9th cerebellar lobule	42	9.20	<	70	4.07	0.030	0.236
Dentate gyrus dorsal	50	10.76	<	88	13.00	0.037	0.189
Habenula n.	13	1.96	<	22	3.61	0.041	0.184
2nd cerebellar lobule	73	9.15	<	103	8.38	0.041	0.193
Inferior colliculus	110	13.26	<	151	12.33	0.041	0.193
Fastigial n. cerebellum	5	1.42	<	8	1.28	0.043	0.201
Subiculum dorsal	21	4.15	<	40	7.34	0.045	0.169
Retrosplenial caudal ctx	101	15.67	<	159	25.94	0.045	0.164
Medial geniculate	14	3.61	<	29	5.33	0.050	0.174
Dorsal medial striatum	62	9.68	<	109	18.61	0.050	0.150

Ave, Average; SE, Standard error; *P* val, Probability value; ω Sq, Omega square; n., nucleus; Ctx, cortex.

**Fig. 2:**
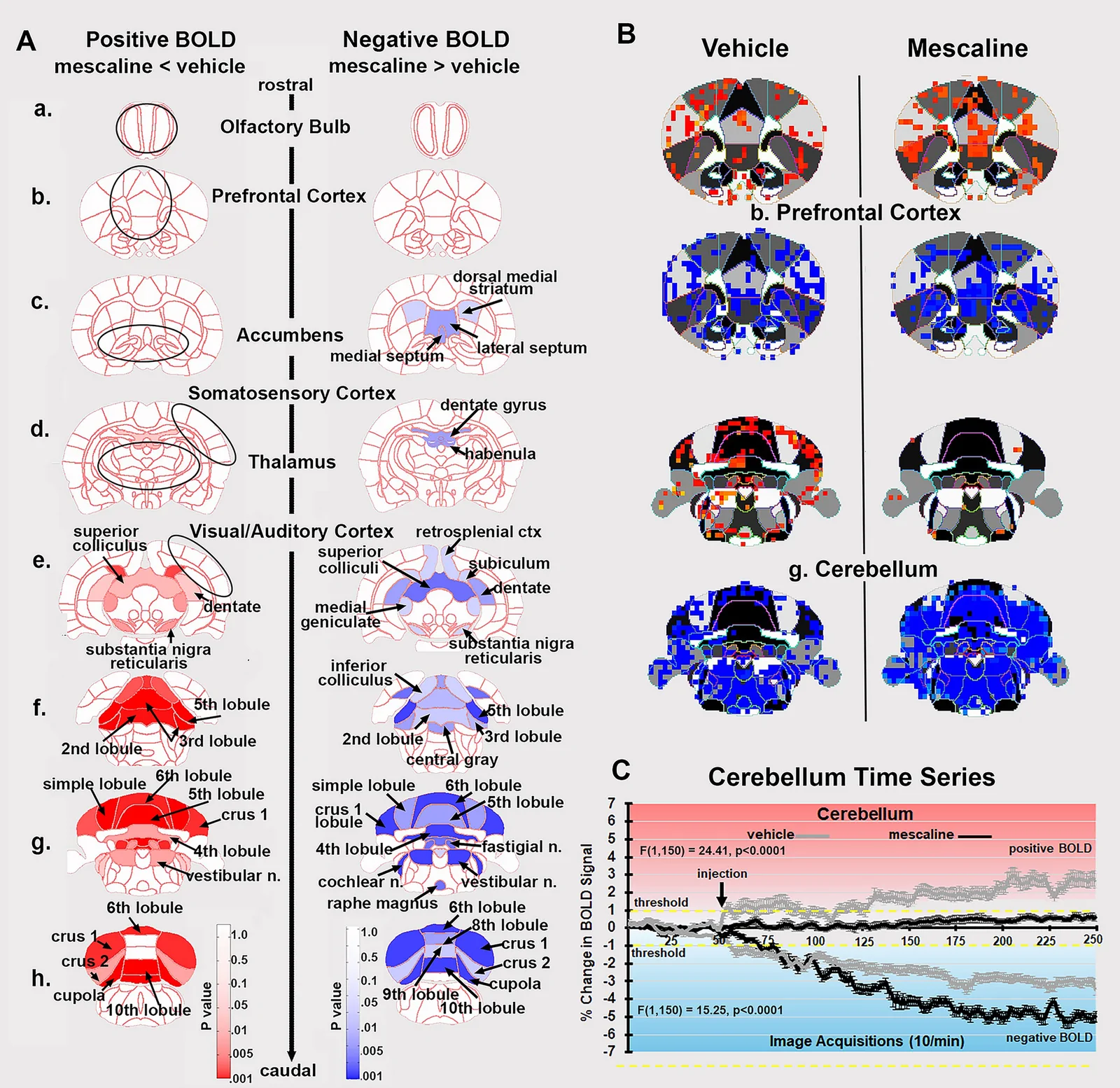
Activation maps and cerebellum time course. The anatomical location of the brain areas listed in Tables 1 and 2. are presented as 2D statistical heat maps (**A**). The coronal sections are labeled (a.) through (h.) and arranged from rostral (top) to caudal (bottom). Areas with changes in positive BOLD are shown in red and indicate they are significantly less than the vehicle. Areas with changes in negative BOLD are shown in blue and indicate they are significantly greater than the vehicle. **B** shows that average positive and negative BOLD voxels (blue and red squares for vehicle and mescaline as heat maps focused on brain slices from rows b. prefrontal cortex, and g. cerebellum. **C** is a time series showing the positive (red shading) and negative (blue shading) change in BOLD signal in the cerebellum in response to vehicle (gray) and mescaline (black). A two-way ANOVA showed a significant increase in vehicle over mescaline (*F =* 24.41, *P <* 0.0001) for positive BOLD and a significant decrease of mescaline over vehicle for negative BOLD (*F =* 15.25, *P <* 0.0001). The intervals along the X axis are image acquisitions collected at 10/min over the 25 min scanning session for a total of 250 images. Injections were given at 5 min or at 50 image acquisitions (arrow). The yellow lines represent 1 % threshold for background noise in awake animal imaging.

The brain areas listed in Tables 1 and 2 are almost exclusively associated with the cerebellum. Fig. 2A shows the anatomical location of the brain areas listed in these tables presented as 2D statistical heat maps. The coronal sections are labeled (a.) through (h.) and arranged from rostral (top) to caudal (bottom). Areas with changes in positive BOLD VoA are shown in red and indicate they are significantly less than the vehicle. Areas with changes in negative BOLD VoA are shown in blue and indicate they are significantly greater than the vehicle. Brain sections (a. & b.) show the absence of any significant changes with mescaline treatment to the olfactory bulbs and prefrontal cortex (ellipses). For positive BOLD VoA, this is also true for the accumbens, somatosensory cortices, thalamus, and visual/auditory cortices (sections c.−e.). Mescaline enhances negative BOLD VoA in striatum and septum (c.) and hippocampal areas dentate and subiculum (d. and e.). Starting at section (e.) and continuing to the most caudal brain section (h.), there is an overlap in brain areas showing the inverse relationship between mescaline-induced positive and negative BOLD VoA. The superior colliculus, medial geniculate and substantia nigra reticularis (e.), inferior colliculus, cerebellum, and vestibular nucleus. (f. – h.)

The absence of any significant differences between mescaline and vehicle for both positive and negative BOLD VoA in sections a.– d. does not imply the absence of a BOLD signal. Fig. 2B shows that average positive and negative BOLD voxels for vehicle and mescaline as heat maps focused on brain slices from rows (b.) prefrontal cortex, and (g.) cerebellum. Note the comparable level of positive and negative BOLD voxels for vehicle and mescaline at level (b.) for the prefrontal cortex, but the large distinction between experimental conditions and BOLD signal at level (g.) for the cerebellum. Additionally, cerebellar activity exhibited a distinct temporal pattern, as depicted in the cerebellum time series shown in Fig. 2C. This time-series analysis provides an alternative perspective on the data, displaying the percentage change in BOLD signal over time. Vehicle data are shown in gray, mescaline data in black, and a yellow baseline marks the threshold for significant changes in awake-animal imaging. A repeated measures two-way ANOVA showed a significant treatment effect between vehicle and mescaline for positive BOLD [*F =* 24.41, *P <* 0.0001] and a significant treatment effect for negative BOLD [*F =* 15.25, *P <* 0.0001]. Notably, mescaline induced a sustained increase in negative BOLD without any increase in positive BOLD, mirroring the VoA data.

### Functional Connectivity

**Fig. 3:**
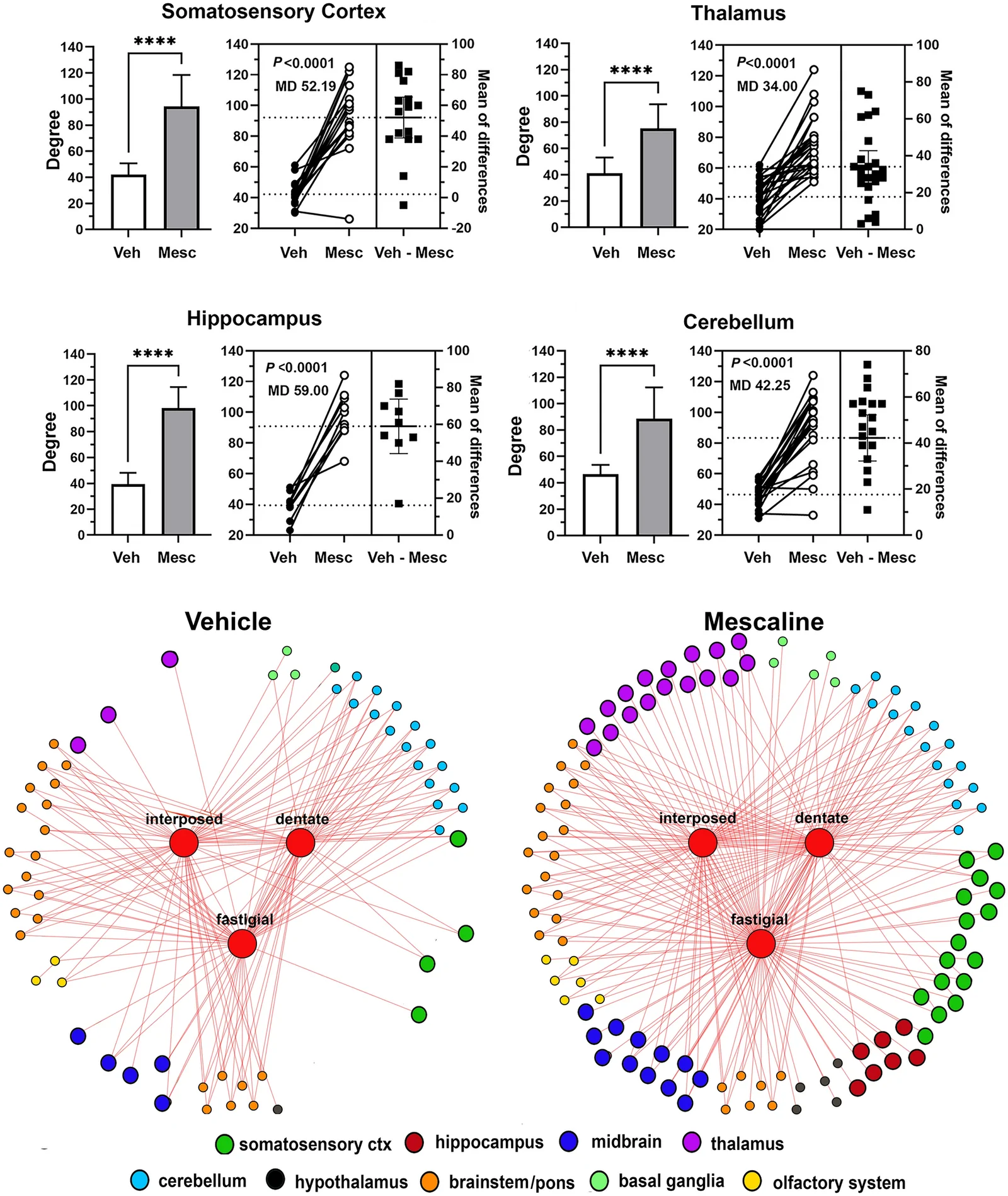
Functional connectivity. Shown in **A** are bar graphs (mean ± SD) and estimation plots for four brain regions that presented with significant changes in the connections between nodes, i.e., degrees. The estimation plots show the difference between vehicle (closed circle) and mescaline (open circle) in the individual brain areas that comprise each brain region, and their mean difference (closed squares). Included in each brain region is the *P* value and the average mean difference. Below in **B** are radial maps showing the connections (lines) between the three areas that comprise the deep cerebellar nuclei, e.g., interposed, dentate, and fastigial (large red dots), and surrounded brain areas are color-coded for their brain region. The enlarged circles highlight the brain regions most affected by mescaline.

Shown in Fig. 3 are changes in functional connectivity following mescaline treatment in different regions of the brain (Supplementary Data File-Brain Regions). For each brain region, there are bar graphs (mean ± SD) together with the individual differences and mean difference (MD) for individual brain areas that comprise the brain region. For example, the hippocampus is comprised of nine areas: dorsal and ventral subiculum, dentate, CA1, CA3, and a single CA2. Comparing vehicle treatment (black circles) to mescaline (white circles) shows a highly significant (*P <* 0.0001) increase in the number of degrees or connections. All brain areas show an increase with mescaline, with a mean difference of 59.00. This hyperconnectivity is observed across all brain regions. In the somatosensory cortex, only the rostral retrosplenial cortex showed a decrease in connectivity. Shown below are radial maps depicting the connections to the three deep cerebellar nuclei (red), e.g., fastigial, dentate, and interposed. The connecting nodes are color-highlighted for different brain regions. For example, nodes in purple identify brain areas in the thalamus, while those in green identify brain areas in the somatosensory cortex. A color code is provided at the bottom of the figure. These maps provide a striking visual presentation of the hyperconnectivity observed with mescaline treatment. The major differences in regional connectivity are highlighted by the larger circles. Note that the thalamus (purple), somatosensory cortex (green), midbrain (blue), and hippocampus (dark red) have greatly enhanced connectivity with the cerebellar nuclei following mescaline exposure. Indeed, the hippocampus has no connection to the cerebellar nuclei but connects to seven of the nine hippocampal areas with mescaline (e.g., dorsal and ventral CA3, dorsal CA1, dorsal and ventral dentate, ventral subiculum, and CA2) (Fig. 3).

**Fig. 4:**
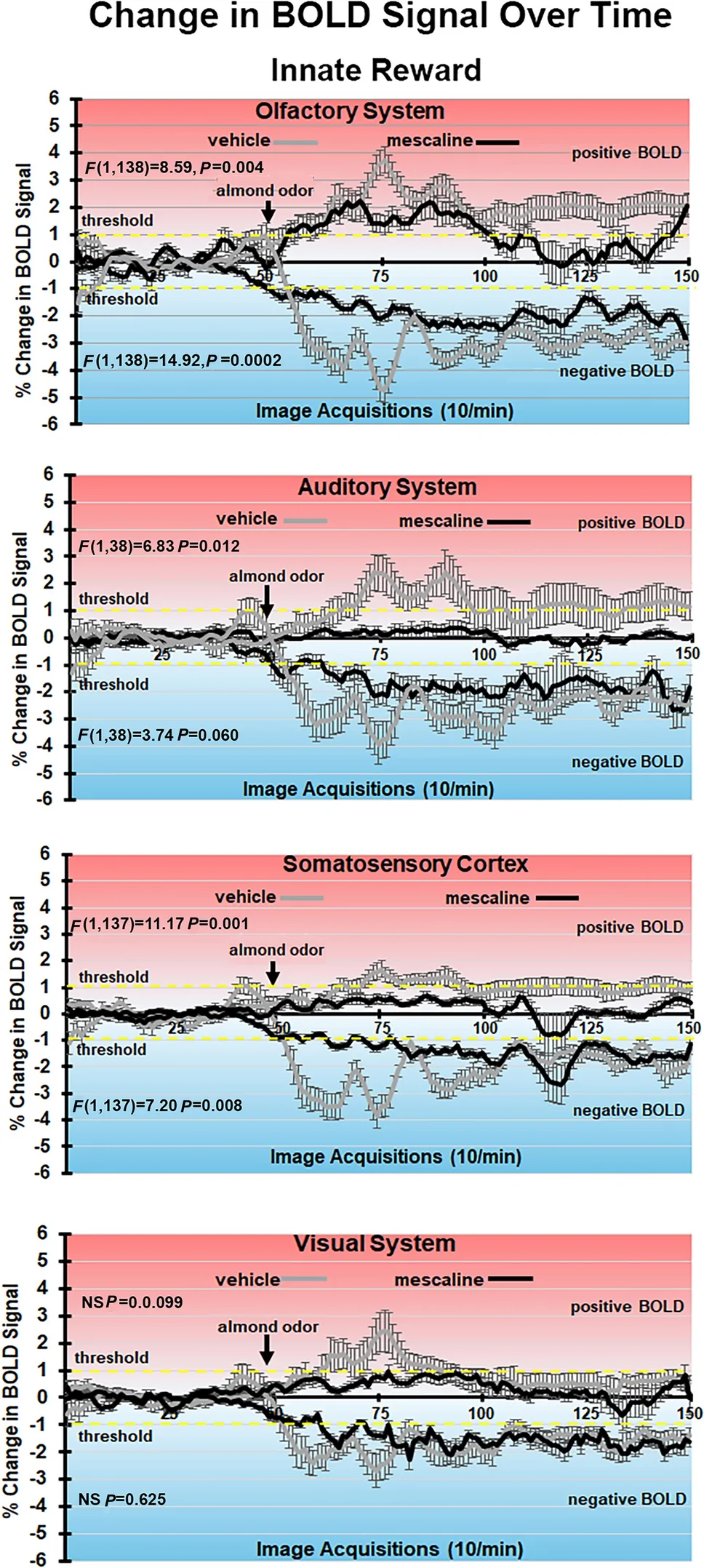
BOLD signal over time for reward. Shown are time course plots for the change in BOLD signal in response to reward (almond odor) for the sensory modalities of smell, hearing, touch, and sight. Description is the same as Fig. 2C.

### Acoustic Startle and Pre-Pulse Inhibition

**Fig. 5:**
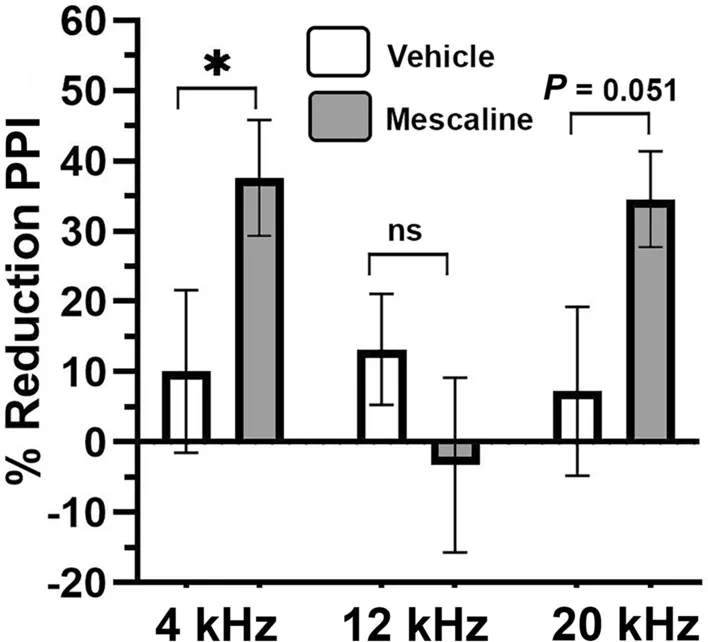
Prepulse inhibition. Shown is the percentage reduction in PPI at 4, 12, and 20 kHz startle frequencies with vehicle (white) and mescaline (gray) treatment. An independent sample t-test showed mescaline significantly (*P =* 0.037) reduced startle amplitude at 4 kHz, with a trend toward significance (*P* = 0.05) at 20 kHz.

Analysis of acoustic startle responses revealed complex frequency-dependent effects of mescaline on sensory gating. Two-way ANOVA identified a nonsignificant Treatment Effect (*F =* 2.97, *P =* 0.0.091), a nonsignificant Frequency Effect (*F =* 2.48, *P =* 0.095), but a significant Treatment x Frequency interaction (*F =* 3.874, *P =* 0.028) and medium effect size (ω^2^ = 0.098). The significant interaction reflects the divergent effects of mescaline across different acoustic intensities. An independent sample *t*-test showed that mescaline significantly reduced the startle amplitude of 4 kHz (*P =* 0.037) and a trend for significance of 20 kHz (*P =* 0.051), but the comparison between vehicle and mescaline was not significant for 12 kHz (Fig. 5). The 4 kHz and 20 kHz intensities enhanced mescaline PPI by +27.6% and +27.3%, respectively, compared to vehicle treatment. The 12 kHz frequency impaired mescaline PPI by −16.4% relative to vehicle. This created an inverted U-shaped frequency response profile, indicating that mescaline's effects on acoustic gating are entirely dependent on the spectral properties of the acoustic stimulus rather than representing a global enhancement or impairment of sensory filtering.

### Odor Provocation for Reward

Following rsFC, approximately 35 min post-mescaline treatment, rats were imaged for changes in brain activity in response to the rewarding stimulus of almond odor. Table 3 is a truncated list of brain areas (mean ± SE) that show a significant change in positive VoA in response to the smell of almond. Significant differences in positive activation were observed in 48/169 brain areas. The brain areas are ranked in order of their significance using a critical value of *P <* 0.05. Shown are the *P*-values and effect size given as omega square (Ω Sq). The false discovery rate (FDR) was *P =* 0.055. Note that in all cases, vehicle-treated animals had a significant increase in positive VoA while mescaline treatment essentially prevented any activation. In Table 4, 32/169 areas displayed a significant change in negative VoA, with an FDR *P =* 0.037. Again, vehicle-treated rats present with significant negative BOLD exceeding mescaline-treated rats across all brain areas.

**Table 3:** Reward positive BOLD volume of activity.

Brain area	Vehicle			Mescaline		*P* val	ω Sq
	Ave	SE		Ave	SE		
Dentate gyrus ventral	6	2.76	>	0	0.00	0.002	0.463
Medial geniculate	4	1.25	>	0	0.10	0.005	0.014
CA3 dorsal	6	3.34	>	0	0.00	0.006	0.016
Habenula n.	5	1.68	>	0	0.40	0.007	0.331
5th cerebellar lobule	26	9.37	>	3	2.89	0.009	0.315
Periaqueductal gray thalamus	23	9.91	>	2	1.72	0.015	0.264
Visual 1 ctx	12	5.07	>	1	0.71	0.015	0.025
Ventral subiculum	6	2.75	>	1	0.61	0.016	0.258
Subiculum dorsal	6	2.99	>	0	0.22	0.016	0.015
Paraventricular n.	1	0.78	>	0	0.00	0.017	0.016
Ventral pallidum	2	1.52	>	0	0.00	0.017	0.253
Anterior thalamic nuclei	5	2.43	>	0	0.00	0.017	0.252
Central gray	3	1.85	>	0	0.00	0.017	0.252
Dorsal medial striatum	18	10.80	>	0	0.00	0.017	0.010
Medial dorsal thalamic n.	3	1.47	>	0	0.00	0.017	0.252
Medial preoptic area	3	1.51	>	0	0.00	0.017	0.252
Parafascicular thalamic n.	8	4.03	>	0	0.00	0.017	0.252
1st somatosensory ctx jaw	38	19.93	>	0	0.00	0.017	0.252
Cochlear n.	2	0.96	>	0	0.00	0.017	0.252
Ventral medial striatum	7	3.71	>	0	0.00	0.017	0.252
1st somatosensory ctx upper lip	22	11.81	>	0	0.30	0.017	0.008
Retrosplenial caudal ctx	43	14.34	>	7	4.59	0.017	0.032
Reticular n. midbrain	15	6.24	>	1	0.45	0.018	0.247
Lemniscal n.	7	2.16	>	1	0.71	0.020	0.237
Anterior cingulate area	46	22.14	>	4	2.36	0.021	0.232
6th cerebellar lobule	6	3.74	>	0	0.20	0.022	0.228
2nd somatosensory ctx	7	4.43	>	0	0.30	0.022	0.228
CA1 dorsal	11	6.64	>	0	0.27	0.025	0.014
Lateral posterior thalamic n.	7	3.57	>	0	0.50	0.027	0.206
Simple lobule cerebellum	13	6.55	>	1	0.43	0.031	0.029
Crus 1 of ansiform lobule	12	4.87	>	1	0.52	0.036	0.181
Inferior colliculus	36	11.77	>	9	4.19	0.038	0.177
Paraventricular n.	0	0.24	>	0	0.00	0.044	0.164
Raphe linear	1	0.44	>	0	0.00	0.044	0.164
Central amygdaloid n.	3	1.95	>	0	0.00	0.044	0.013
Claustrum	3	1.70	>	0	0.00	0.044	0.163
Dorsal lateral striatum	17	10.12	>	0	0.00	0.044	0.004
Inferior olivary complex	1	0.80	>	0	0.00	0.044	0.163
Lateral dorsal thalamic n.	1	0.46	>	0	0.00	0.044	0.163
Medial mammillary n.	2	1.03	>	0	0.00	0.044	0.163
Posterior hypothalamic area	3	1.85	>	0	0.00	0.044	0.163
Premammillary n.	1	0.53	>	0	0.00	0.044	0.163
Reuniens n.	2	1.67	>	0	0.00	0.044	0.163
Reticular n.	4	2.76	>	0	0.00	0.044	0.163
Ventral anterior thalamic n.	1	1.09	>	0	0.00	0.044	0.006
Ventrolateral thalamic n.	3	2.19	>	0	0.00	0.044	0.017
3rd cerebellar lobule	9	4.56	>	1	0.69	0.050	0.152
Flocculus cerebellum	2	0.88	>	0	0.10	0.050	0.151

Ave, Average; SE, Standard error; *P* val, Probability value; ω Sq, Omega square; n., nucleus; Ctx, cortex.

**Table 4:** Reward negative BOLD volume of activation.

Brain area	Vehicle			Mescaline		*P* val	ω Sq
	Ave	SE		Ave	SE		
Reticular n. pons	106	20.04	>	16	6.94	0.001	0.555
Crus 2 of ansiform lobule	47	10.74	>	10	6.78	0.002	0.475
Raphe obscurus n.	3	0.55	>	0	0.30	0.002	0.482
Solitary tract n.	22	4.32	>	4	1.87	0.002	0.455
8th cerebellar lobule	22	8.91	>	4	4.61	0.002	0.489
Pedunculopontine tegmental n.	4	0.94	>	0	0.22	0.004	0.414
1st sensory n. trigeminal	45	9.63	>	12	3.56	0.006	0.345
Pontine nuclei	32	9.91	>	8	5.05	0.006	0.348
2nd cerebellar lobule	58	12.81	>	18	7.30	0.007	0.334
Root of trigeminal nerve	42	7.52	>	15	6.43	0.008	0.315
Flocculus cerebellum	14	2.70	>	5	1.64	0.009	0.321
Parvicellular reticular n.	67	18.24	>	15	5.58	0.011	0.287
Medial septum	3	1.02	>	0	0.40	0.011	0.316
Vestibular n.	52	9.75	>	18	6.57	0.013	0.281
Raphe magnus	8	2.03	>	1	0.53	0.013	0.322
Pontine reticular n. oral	20	6.54	>	4	1.54	0.014	0.280
Pontine reticular n. caudal	60	16.88	>	16	7.68	0.016	0.262
Crus 1 of ansiform lobule	119	22.01	>	49	22.24	0.020	0.244
10th cerebellar lobule	15	5.34	>	5	3.82	0.022	0.264
Paramedian lobule	42	10.97	>	10	4.91	0.024	0.227
Dorsal paragigantocellular n.	10	2.10	>	4	1.93	0.024	0.224
Cochlear n.	14	3.34	>	6	2.84	0.026	0.236
CA1 hippocampus ventral	30	9.93	>	9	5.00	0.031	0.228
9th cerebellar lobule	21	8.40	>	5	4.27	0.031	0.216
4th cerebellar lobule	42	10.77	>	17	6.66	0.033	0.195
Paraflocculus cerebellum	99	17.92	>	49	16.79	0.033	0.191
Infralimbic ctx	22	6.77	>	9	5.33	0.041	0.172
Anterior amygdaloid n.	1	0.34	>	0	0.00	0.044	0.214
trapezoid body	9	2.67	>	3	1.82	0.046	0.199
Facial n.	11	3.05	>	3	1.81	0.046	0.169
Sub coeruleus n.	18	5.31	>	6	2.03	0.047	0.183
Parabrachial n.	19	4.97	>	8	3.04	0.049	0.162

Ave, Average; SE, Standard error; *P* val, Probability value; ω Sq, Omega square; n., nucleus; Ctx, cortex.

Hence, mescaline significantly alters the perception of reward. While mescaline alters the olfactory perception of the environment, does it also affect the sensitivity of other sensory modalities? Fig. 4 shows the change in BOLD signal over time for olfaction, hearing, vision, and sensation for reward. Each time course diagram shows the positive (red) and negative (blue) BOLD signal change for mescaline (black line) and vehicle (gray line) treated rats. 150 images were acquired over the 15-minute imaging session. Odor was applied at 5 min (arrow). Each acquisition is the mean ± SE of vehicle and mescaline, combining the data from the brain areas associated with that specific sensory modality. The data for the visual system came from the visual cortices V1 and V2 and the lateral geniculate. The data for the auditory system came from the auditory cortex and the medial geniculate. The somatosensory system was comprised of the primary somatosensory cortices for the barrel field, lips, shoulder, fore and hindlimbs. The olfactory system was comprised of the olfactory bulb, the anterior olfactory nucleus, the piriform cortices, and the entorhinal cortex. The significant differences between treatments were assessed with a two-way repeated measures ANOVA. The *F* value and *P* values for positive and negative differences in BOLD signal over time are provided in each figure. Differences that were not significant (NS) are noted. The yellow lines marking ± 1% are the threshold for signal above noise when imaging awake rats. When looking at the innate response to the reward of almond odor in the olfactory system, mescaline was significantly less than vehicle for both positive and negative BOLD. This was also true for the somatosensory cortex and the auditory system (trend toward significance, *P* = 0.060 for negative BOLD). There were no significant differences between treatments for the visual system.

## Discussion

This study presents the first comprehensive neuroimaging characterization of mescaline's acute effects on brain function, revealing a distinctive neurobiological profile that sets it apart from other classical psychedelics. While previous human studies have documented mescaline's behavioral and physiological effects—including visual distortions, altered time perception, autonomic changes, and 5-HT2a receptor-mediated mechanisms [6, 35, 36] — the absence of neuroimaging data has left critical gaps in understanding its neural substrates. Our findings demonstrate that mescaline produces a unique cerebellar-selective activation pattern fundamentally different from the cortical and thalamic effects reported for LSD [37, 38] and the cortico-striato-thalamic activation seen with psilocybin [39].

The earliest and only previous neuroimaging study of mescaline, conducted by Hermle *et al.* using SPECT in twelve volunteers, reported increased right frontal blood flow but decreased flow to the right thalamus and superior-temporal cortex [40]. Our rat data reveal a strikingly different pattern, with minimal forebrain involvement and pronounced cerebellar effects. This discrepancy may reflect species differences, methodological variations, or the superior spatial and temporal resolution of our awake animal BOLD fMRI approach compared to SPECT imaging.

### Cerebellar-Selective Effects and Functional Disconnection

The most striking finding of this study is mescaline's selective impact on the cerebellum, characterized by simultaneous decreases in positive BOLD VoA and increases in negative BOLD VoA across multiple cerebellar regions. This pattern suggests the cerebellum has a unique sensitivity to mescaline in a BOLD provocation paradigm that is not shared by forebrain structures, e.g., thalamus, sensorimotor cortex, prefrontal cortex, basal ganglia, a neurobiological signature not reported for other classical psychedelics. This pattern suggests a fundamental dysregulation of local cerebellar processing**,** potentially indicating a shift in the balance of excitation and inhibition within cerebellar microcircuits, such as altered Purkinje cell output or granule cell layer activity. While LSD primarily affects the olfactory system, prefrontal cortex, thalamus, and hippocampus [37, 38], and psilocybin engages cortico-striato-thalamic circuits [39], mescaline appears to specifically target cerebellar processing networks.

The cerebellum's role extends far beyond motor coordination, encompassing cognitive functions including attention, memory, language, and emotional regulation [41]. With over half the brain's neurons densely packed in its granule cell layer and communication channeled through just three output pathways—the deep cerebellar nuclei—the cerebellum is optimally positioned to process massive amounts of information and distill it into refined modulatory signals [42, 43]. Our connectivity analysis shows the cerebellum develops extensive hyperconnectivity with regions including the hippocampus, thalamus, somatosensory cortex, and midbrain. This pattern of local hypoactivation coupled with global hyperconnectivity may reflect a two-stage disruption: an initial suppression or desynchrony of local cerebellar computation, followed by a compensatory or maladaptive loss of network segregation, leading to aberrant communication with forebrain structures [39, 44, 45].

### The Cerebellum as a Dysregulated Sensory Gate

Our findings align with emerging theories of cerebellar function that position it as a critical sensory filtering mechanism. Devor's "Great Gate" posits that the cerebellum, in concert with the inferior olivary complex, functions as a predictive filter, distinguishing expected from unexpected sensory inputs [46]. Under normal conditions, climbing fiber inputs signal prediction errors (novelty), while the cerebellum provides inhibitory feedback to suppress anticipated, redundant information. This predictive coding framework positions the cerebellum as a sophisticated comparator, essential for filtering noise and amplifying behaviorally relevant signals [47]. Mescaline's disruption of this system, evidenced by cerebellar hypoactivation coupled with hyperconnectivity, may effectively 'open the gate' by impairing the cerebellum's ability to generate precise predictive signals. The local hypoactivation could reflect a disruption in the internal model or a decoupling of the cerebellar cortex from its nuclei. Consequently, the dysregulated cerebellum may fail to filter and refine ascending sensory information, instead flooding connected cortical and subcortical regions with unprocessed or poorly contextualized sensorimotor data. This proposed mechanism, a dysregulated sensory filter, provides a parsimonious explanation for the characteristic perceptual alterations and sensory flooding reported in psychedelic experiences. The hyperconnectivity we observed, particularly between the cerebellum and previously disconnected regions like the hippocampus, suggests mescaline transforms the cerebellum from a selective, predictive filter into an untethered broadcaster of unfiltered information, potentially driving altered states of consciousness through a cascade of downstream integration failures.

This framework is further supported by our frequency-selective acoustic gating findings. The inverted U-shaped effect on PPI indicates that mescaline does not cause a global impairment of sensory filtering but rather recalibrates the gain of specific sensory channels**.** This is consistent with a disruption of the cerebellum's role in tuning the precision (or gain) of sensory predictions within a predictive coding hierarchy. The convergence of our imaging and behavioral data suggests mescaline fundamentally alters the cerebellar contribution to sensory prediction error signaling — a mechanism that warrants direct testing in future studies combining high-resolution fMRI with computational modeling.

### Frequency-Selective Acoustic Processing

Perhaps the most unexpected finding was mescaline's frequency-dependent effects on acoustic gating. Unlike the global PPI disruption typically reported for psychedelics in rodents [48, 49], mescaline enhanced acoustic gating at low (4 kHz) and high (20 kHz) intensities while impairing it at an intermediate frequency (12 kHz). This creates an inverted U-shaped response profile that suggests mescaline fundamentally alters the relationship between stimulus frequency and sensory filtering rather than simply enhancing or disrupting gating globally.

### Implications for Schizophrenia Research

The cerebellar dysfunction observed with mescaline bears striking similarities to abnormalities reported in schizophrenia, providing new insights into psychedelic-psychosis relationships. Schizophrenia involves structural cerebellar changes, including reduced gray matter volume [50], altered functional connectivity with sensory and limbic regions [51], and hypoactivation in motor and cognitive processing areas [52]. The involvement of serotonergic signaling in both schizophrenia pathology [53, 54] and cerebellar function [55] suggests shared neurobiological substrates.

Our findings support emerging theories implicating cerebellar dysfunction in schizophrenia symptoms through disrupted cerebello-thalamo-cortical circuits (CTCCs) [56–59]. The hyperconnectivity between cerebellar and cortical regions we observed with mescaline mirrors connectivity patterns seen in first-episode schizophrenia patients [51], suggesting psychedelics may provide valuable models for understanding psychotic states. However, unlike the cognitive deficits characteristic of schizophrenia, psychedelic experiences often involve enhanced creativity and novel associations, highlighting important distinctions between pathological and pharmacologically induced altered states.

The observed pattern of cerebellar hyperconnectivity mirrors emerging models of psychosis, where a failure of sensory filtering and prediction error signaling is thought to lead to a flood of uncensored sensory information and aberrant salience [60]. Mescaline's effects thus provide a pharmacological model of how cerebellar dysregulation within predictive coding circuits may contribute to altered perceptual states, bridging neurophysiology with phenomenology.

### Selective Sensory Modulation and Reward Processing

Mescaline's effect on reward processing suggests a disruption in the sensory processing of positive salient cues. The sensory modality analysis revealed that mescaline's effects extend beyond olfaction to include somatosensory and auditory systems, while largely sparing visual processing. This pattern differs from human reports of prominent visual distortions with mescaline [61] and may reflect species differences in sensory system organization or the specific doses and timepoints examined.

### Clinical and Therapeutic Implications

These findings have important implications for mescaline's potential therapeutic applications. The unique cerebellar targeting suggests mescaline might be particularly effective for conditions involving cerebellar dysfunction, including certain movement disorders, cognitive impairments, and psychiatric conditions with cerebellar involvement. The frequency-selective acoustic gating effects suggest mescaline might have applications in treating auditory processing disorders or conditions involving sensory filtering deficits. However, the complexity of these effects underscores the need for careful dose optimization and individual monitoring in clinical applications.

### Limitations and Future Directions

Several limitations warrant consideration. The awake rat model, while providing unprecedented spatial and temporal resolution, may not fully capture the subjective experiential aspects of psychedelic effects. Species differences in brain organization, particularly cerebellar-cortical connectivity patterns, may limit direct translation to humans. The single dose tested (50 mg/kg) represents one point on the dose-response curve, and lower or higher doses might reveal different patterns of activation. Future studies should examine dose-dependent effects, longer timepoints to capture potential neuroplasticity changes, and direct comparisons with other psychedelics under identical experimental conditions. Investigation of the molecular mechanisms underlying cerebellar selectivity, including receptor distribution patterns and downstream signaling cascades, will be crucial for understanding mescaline's unique profile.

An inherent limitation of psychopharmacological imaging is the impossibility of completely dissociating direct neural effects from brain responses secondary to peripheral physiological changes. Mescaline produces autonomic effects, including increased heart rate and blood pressure in humans [7], which themselves alter cerebral blood flow and BOLD signal. While our vehicle control accounts for non-specific effects of the imaging environment, it cannot distinguish between: (1) direct 5-HT2A receptor-mediated neural effects, (2) central neural responses to peripheral physiological changes, and (3) neurovascular coupling alterations. This represents a fundamental challenge in drug imaging research, as the 'drug state' is inherently a holistic physiological condition.

A specific limitation of our study is the absence of concurrent physiological monitoring (respiration rate, heart rate, blood gases) during mescaline exposure. When we established the awake MRI protocol in rats, we documented the effects of acclimation on autonomic arousal, stress hormones, cerebral blood flow, and BOLD signal [25]. However, in this case, we did not monitor whether mescaline itself alters the stress response in ways that could contribute to the observed activation patterns. Future studies should incorporate concurrent respiratory and cardiac monitoring to enable physiological noise correction and to better dissociate neural from cardiovascular contributions to observed BOLD signal changes.

Additionally, recent work by Epp *et al.* demonstrates that BOLD signal changes can oppose underlying oxygen metabolism in approximately 40% of cortical voxels, with discordance particularly prominent in the default mode network [62]. This finding challenges simplistic interpretations of BOLD signals as direct proxies for neural activity and indicates that our BOLD-based interpretations require validation through multimodal approaches combining fMRI with direct neural measurements (electrophysiology, calcium imaging) or quantitative metabolic imaging. The extent to which cerebellar BOLD responses to psychedelics exhibit concordant versus discordant neurovascular coupling remains an important open question.

Despite these important caveats, multiple lines of evidence support a neural, rather than purely hemodynamic, origin of our findings. First, our negative BOLD responses closely parallel findings from human fMRI studies with psilocybin, LSD, and other classical psychedelics [39, 63]. In these human studies, reduced BOLD signal has been validated against EEG and MEG data, confirming neural origins. The consistency between our rodent findings and these well-validated human studies strongly supports a neural interpretation. Second, if the BOLD changes were primarily due to systemic cardiovascular effects, we would expect uniform changes across all brain regions. Instead, we observe highly region-specific patterns affecting the hindbrain with little or no effect on forebrain positive and negative BOLD signal. This regional specificity—particularly the cerebellar selectivity—argues against purely global autonomic effects. Third, our previous studies with LSD [16] and psilocybin [17] in the same awake rat preparation showed similar negative BOLD patterns that correlated with behavioral effects, further supporting neural origins.

In summary, while we cannot completely exclude contributions from autonomic and neurovascular mechanisms, the regional selectivity, cross-psychedelic consistency, behavioral correlations, and validation against human electrophysiological data provide reasonable grounds for interpreting our BOLD findings as reflecting altered neural activity patterns, with the explicit acknowledgment that multimodal validation studies are needed to definitively resolve this question.

## Conclusions

This study establishes mescaline as having a distinctive neurobiological profile characterized by cerebellar-selective hypoactivation coupled with global hyperconnectivity. The cerebellum emerges as a critical hub in psychedelic action, functioning as a dysregulated sensory filter that transforms normal predictive processing into untethered information broadcasting. The frequency-selective acoustic gating effects reveal novel mechanisms of sensory processing modulation that distinguish mescaline from other psychedelics. These findings provide the neurobiological foundation for understanding mescaline's unique therapeutic potential while advancing our broader understanding of how psychedelics alter consciousness through selective neural circuit modulation.

## Supplementary Information

Below is the link to the electronic supplementary material.

Supplementary file1 (XLSX 12 KB)

Supplementary file2 (JPG 598 KB)

Supplementary file3 (JPG 330 KB)

Supplementary file4 (XLSX 80 KB)
